# Current Advancement of Respiratory Stability Time-Guided Heart Failure Management

**DOI:** 10.3390/jcm14176182

**Published:** 2025-09-01

**Authors:** Teruhiko Imamura

**Affiliations:** Second Department of Internal Medicine, University of Toyama, Toyama 930-0194, Japan; te.imamu@gmail.com

**Keywords:** telemonitoring, sleep monitoring, digital health, heart failure, hemodynamics

## Abstract

Heart failure (HF) remains a global health challenge with high rates of hospitalization and mortality, particularly among the elderly. Many episodes of worsening HF occur before symptoms arise, underscoring the need for sensitive monitoring tools. Respiratory Stability Time (RST) is a novel index that quantifies the duration of stable respiration during sleep, reflecting pulmonary congestion and circulatory status. RST can be measured continuously and non-invasively using a contactless under-mattress sensor. Observational cohort studies show that low RST predicts poor prognosis, while its improvement parallels recovery from decompensation. Importantly, recent prospective multicenter observations involving 100 patients demonstrated that sustained RST decline often precedes HF readmission, probably enabling early intervention. A multicenter trial (ITMETHOD-HF III), involving 80 patients, is currently testing whether RST-guided therapy can reduce HF readmissions. RST might substantially enhance current HF management by enabling us to provide proactive therapeutic intervention, though further validation is warranted.

## 1. Heart Failure Pandemic

Heart failure (HF) has emerged as a global public health challenge with profound clinical, societal, and economic implications [1]. It is estimated that over 64 million people worldwide are living with HF, a number that continues to rise in parallel with population aging and improved survival from cardiovascular events. In developed nations, HF is a leading cause of hospitalization among individuals aged 65 years and older, with a 5-year mortality rate comparable to that of some cancers [2]. Despite advances in pharmacological and device-based therapies, the burden of HF remains substantial, largely due to high rates of hospitalization and re-hospitalization, especially in the elderly [3,4].

In Japan, the demographic profile is particularly notable for its rapid aging, with over 29% of the population aged 65 years or older. This trend has contributed to a continuous increase in the number of HF patients, a phenomenon often referred to as the “HF pandemic” [5]. The prevalence of HF in Japan is estimated to exceed 1 million, and projections suggest that this number will reach 1.3 million by 2030 [6]. The Japanese Circulation Society has reported that HF-related hospitalizations have also increased accordingly, with high readmission rates observed within 6 months of discharge, particularly among elderly patients with multiple comorbidities, such as chronic kidney disease, atrial fibrillation, diabetes mellitus, and frailty [7].

Mortality in HF patients remains unacceptably high despite contemporary guideline-directed medical therapy (GDMT) [8,9]. The 5-year survival rate after an initial hospitalization for HF is approximately 50% in both Western countries and Japan. Furthermore, the increasing proportion of elderly HF patients—many of whom are frail and have preserved ejection fraction—has complicated therapeutic strategies and limited the applicability of certain evidence-based treatments [10].

Over the past two decades, treatment strategies for HF have evolved significantly. The introduction of beta-blockers, mineralocorticoid receptor antagonists, angiotensin receptor–neprilysin inhibitors, and sodium–glucose co-transporter 2 inhibitors has substantially transformed the management of HF with reduced ejection fraction [11]. Device therapies, including implantable cardioverter-defibrillators and cardiac resynchronization therapy, have also improved clinical outcomes in carefully selected populations [12]. Nevertheless, the management of HF with preserved ejection fraction remains challenging, with fewer proven therapeutic options, such as sodium–glucose co-transporter 2 inhibitors [13].

## 2. The Prognostic Significance of HF Re-Hospitalization

Readmission after the index hospitalization for HF is a critical clinical event and a strong predictor of poor prognosis [14]. Many patients are readmitted within a few weeks or months following discharge, and each additional hospitalization tends to worsen long-term outcomes [4]. Repeated episodes of acute decompensation not only indicate disease progression but also lead to cumulative organ damage, increased frailty, and a decline in patients’ quality of life [15].

From a clinical standpoint, recurrent hospitalizations are associated with higher mortality and a greater burden of symptoms [16]. Patients often experience worsening renal function, pulmonary congestion, and reduced physical activity following repeated admissions [17]. Moreover, each re-hospitalization tends to be more severe and harder to reverse than the last, given the progressive nature of chronic HF [18]. For elderly patients, especially those with multiple comorbidities, even a single episode of acute HF can accelerate overall physical and cognitive decline.

In addition to the impact on individual patients, frequent readmissions place a significant burden on healthcare systems, consuming substantial medical resources and increasing healthcare costs [19]. Thus, preventing decompensation before it leads to hospitalization should be a central goal of modern HF management.

## 3. How to Prevent HF Re-Hospitalization

While contemporary GDMT and multidisciplinary care have contributed to outcome improvements, these strategies often rely on reactive approaches—initiated only after symptoms appear. However, many episodes of worsening HF are preceded by subtle physiological changes, such as an increase in intra-cardiac loading [20].

Given the strong association between HF decompensation and adverse clinical outcomes, early detection of worsening physiological status has become a central focus in modern HF management. Which parameters should we monitor to identify the early phase of HF deterioration?

Furthermore, these parameters are not specific to HF. This gap has stimulated the development and adoption of remote monitoring technologies, as mentioned below, which aim to detect early signs of congestion and guide timely intervention before hospitalization becomes necessary.

## 4. CardioMEMS

Several remote monitoring strategies have been introduced over the past two decades (Table 1). Among the most studied is the CardioMEMS system, an implantable pulmonary artery pressure sensor that allows daily transmission of hemodynamic data to clinicians [21]. CardioMEMS has demonstrated clinical benefit in carefully selected patients with HF with reduced or preserved ejection fraction by enabling preemptive adjustment of medication, such as diuretics [22]. However, the system requires an invasive implantation procedure and is primarily used in patients with prior hospitalization, limiting its broader applicability. Furthermore, the system has not been approved and/or reimbursed in many countries, including Japan.

## 5. Thoracic Impedance Monitoring

Another approach, OptiVol, is an intrathoracic impedance monitoring system integrated into implantable pacemakers (Table 1). It estimates pulmonary fluid accumulation by measuring changes in electrical impedance across the chest. While conceptually appealing, the predictive value of OptiVol has been limited by low specificity, leading to frequent false-positive alerts and clinical uncertainty [23,24]. The system is available only to patients who have undergone pacemaker implantation. Consequently, it has not been widely adopted as a stand-alone tool for HF monitoring.

## 6. Remote Dielectric Sensing

Remote dielectric sensing (ReDS) is a non-invasive technology that quantitatively assesses pulmonary fluid levels by measuring the dielectric properties of lung tissue (Table 1) [25,26]. Several studies have suggested that ReDS can enable early detection of subclinical pulmonary congestion, potentially allowing for timely therapeutic interventions before overt HF decompensation occurs [27,28,29]. Its objective nature and ability to detect fluid accumulation even in the absence of symptoms position ReDS as a promising tool for remote HF monitoring, particularly in high-risk patient populations.

Despite these advantages, the widespread adoption of ReDS in routine clinical practice remains limited. Key barriers include the cost and logistical burden of the device, which may not be readily available in all care settings. While ReDS values provide direct information on lung fluid content, their interpretation may be confounded by coexisting pulmonary conditions, such as chronic obstructive pulmonary disease or interstitial lung disease [30]. The system cannot distinguish pulmonary congestion from other lung diseases, such as pulmonary pneumonia.

## 7. Telemonitoring Programs

In addition to device-based monitoring, telemonitoring programs that involve the regular transmission of body weight, blood pressure, and symptom checklists have also been evaluated (Table 1) [31]. Although these programs offer logistical simplicity and broader access, clinical trials have yielded mixed results, with some studies showing limited impact on reducing rehospitalization rates or mortality [32]. This may reflect the subjective nature of symptom reporting and the delayed physiological signals captured by these conventional parameters [33].

Taken together, these technologies have provided valuable insights and laid the foundation for remote management strategies, but their limitations remain evident (Table 1). Most current monitoring systems are constrained by invasiveness, suboptimal sensitivity and specificity, or reliance on patient engagement, which can be particularly unreliable in elderly or frail populations. These shortcomings highlight the need for a non-invasive, passive, and scalable tool—criteria that RST uniquely meets by enabling continuous and objective detection of early HF decompensation [34].

**Table 1:** Comparison of potential procedures for remote monitoring.

Technology	Physiological Parameter	Method	Invasiveness	Predictive Value	Representative Study Outcome	Strengths/Weaknesses
CardioMEMS	Pulmonary artery pressure	Implantable sensor (pulmonary artery)	Invasive (trans-catheter)	High (in selected high-risk patients)	CHAMPION and MONITOR-HF: HF hospitalization ↓ [21,22]	Strength: strong outcome data. Weakness: requires implantation. Sensitivity high, but limited generalizability.
OptiVol	Intrathoracic impedance (fluid accumulation)	Pacemaker-based impedance measurement	Minimally invasive	Limited (low specificity)	SENSE-HF: poor predictive accuracy [24]	Strength: available in pacemaker patients. Weakness: high false positive. Sensitivity low.
Remote dielectric sensing	Lung fluid content	Electromagnetic signal through thorax	Non-invasive	Moderate to high (validated in trials)	ReDS-SAFE HF: early readmission risk ↓ [29]	Strength: quantitative. Weakness: device cost/logistics. Specificity moderate, confounded by lung disease.
Telemonitoring	Symptoms, weight, blood pressure, heart rate	Patient-reported or home-based devices	Non-invasive	Variable (depends on adherence and setting)	TIM-HF2: mortality and HF hospitalization ↓ [33]	Strength: wide accessibility. Weakness: reliance on adherence, subjective data. Sensitivity low.

HF, heart failure.

## 8. What Is Respiratory Stability Time?

Respiratory Stability Time (RST) is a novel physiological indicator that quantifies the stability of respiratory patterns during sleep [35]. It represents the cumulative duration in which a person maintains regular, undisturbed breathing during sleep. In short, a longer RST reflects a state of cardiopulmonary stability, while a shorter RST suggests increased respiratory variability and underlying pathophysiological stress. This measurement has attracted clinical attention as a potential early marker for HF decompensation.

## 9. How to Calculate RST

**Figure 1:**
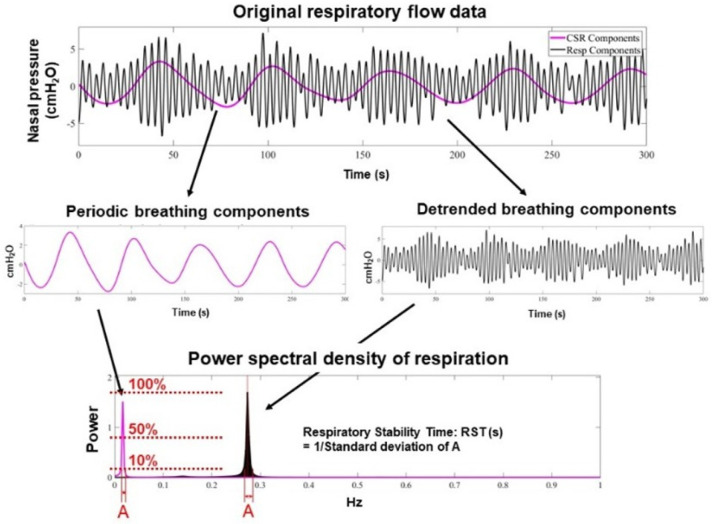
Methodology for RST calculation using spectral analysis [36]. Digitized respiratory signals are analyzed using bandpass filters to extract high- and ultra-low-frequency components. The maximum entropy method is applied to derive the power spectral density. RST is defined as the inverse of the standard deviation of frequency points with power spectral density > 10% in the high-frequency band or >50% in the ultra-low-frequency band, representing the duration of stable respiration.

As summarized in Figure 1, respiratory patterns are digitized and sampled at 16–100 Hz [36]. We focus on two frequency ranges to estimate RST [35]. One range consists of respiratory frequency components obtained from the instantaneous ventilation signal with very high- or low-frequency noise removed via a bandpass filter with cutoff frequencies of 0.08 Hz and 0.6 Hz. The other components constitute a very low-frequency range of respiration as an indicator of periodic respiration power. These data are obtained by tracing the peak of the instantaneous ventilation signal, adjusting the baseline to zero, and applying a bandpass filter with cutoff frequencies of 0.008 and 0.04 Hz. For each 5 min epoch, the maximum entropy method is applied to these respiratory and periodic breathing curves to perform a spectral analysis of each wave [37]. All spectral power is normalized by the power spectral density, or the ratio of the maximum power of the components. This describes how the power of a signal or time series is distributed over frequency. All respiration frequency points with power spectral density > 10% are equally adopted in the standard deviation for the assessment of respiratory instability.

Very low frequency points of the periodic breathing curve are also adopted only if the power spectral density of the very low frequency component is >50% of the maximum power of the respiratory component.

**Figure 2:**
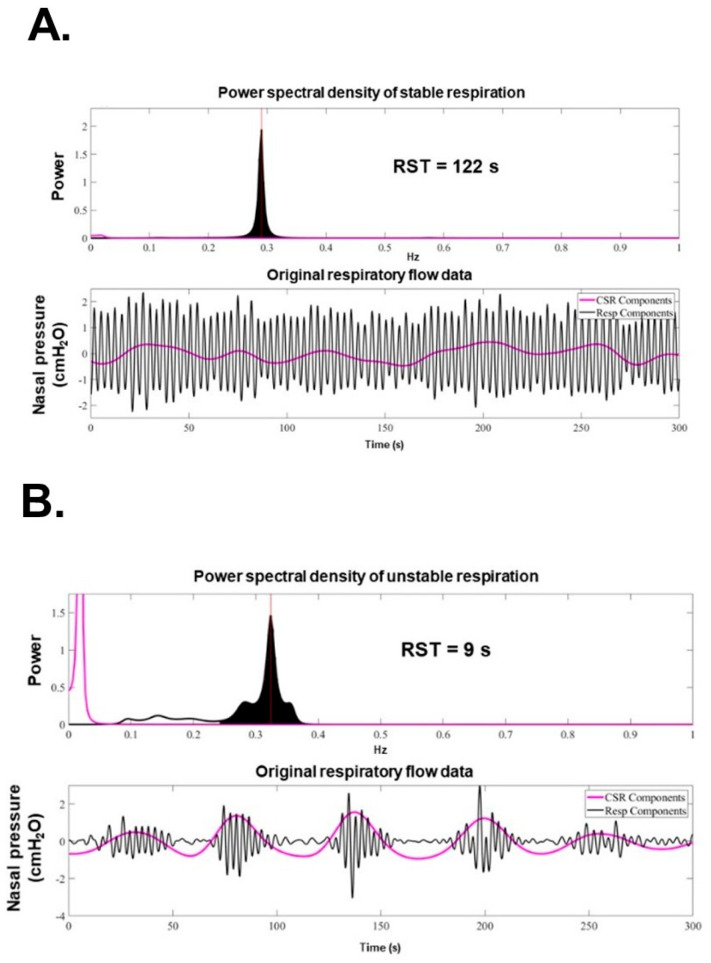
Representative examples of high and low RST during sleep [36]. (**A**) Stable respiration in a compensated patient with RST = 122 s, showing regular respiration and a narrow-band spectral distribution. (**B**) Irregular and periodic respiration in a decompensated patient with RST = 9 s, showing prominent ultra-low-frequency activity (i.e., Chain–Stokes respiration). Spectral and waveform analysis illustrates the association between respiratory irregularity and reduced RST.

RST is defined as the inverse of the standard deviation [35]. Since RST is the inverse of frequency (Hz), it is expressed as a unit of time (s). In other words, it corresponds to the time during which an unchanged and stable breathing pattern is repeated and indicates the stability of breathing. Typical representatives of normal breathing with high RST (RST 122 s) and of abnormal breathing with low RST due to an irregular and periodical respiratory pattern (RST 9 s) are displayed in Figure 2A,B [36].

## 10. Theoretical Physiological Relationship Between RST and HF

**Figure 3:**
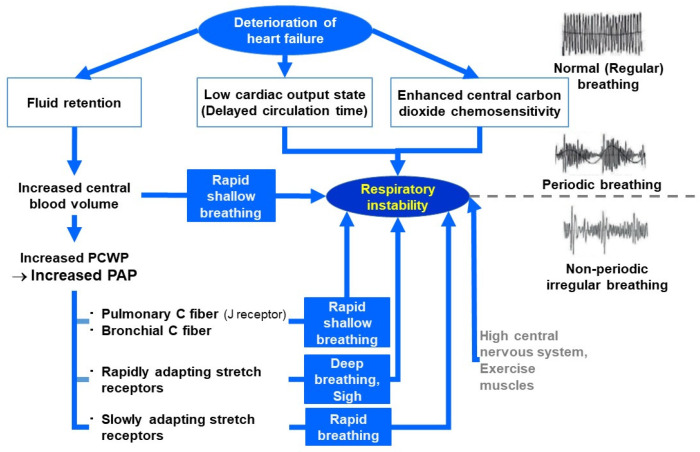
Pathophysiological mechanisms underlying respiratory instability in heart failure. Heart failure decompensation leads to pulmonary congestion, elevated pulmonary artery pressure, and stimulation of pulmonary stretch and C fibers. Simultaneously, low cardiac output and delayed circulation enhance central CO_2_ sensitivity. These factors induce irregular respiratory drive, resulting in abnormal patterns such as periodic breathing, rapid shallow breathing, and sighing, all of which decline RST levels. Reused with permission from Hidetsugu Asanoi.

The physiological rationale behind RST is illustrated in Figure 3, which highlights the dynamic interplay between cardiovascular decompensation and respiratory control [38]. Under stable conditions, particularly during sleep, the respiratory pattern remains regular due to preserved autonomic regulation and adequate cardiac output. However, as HF begins to deteriorate, multiple physiological disturbances emerge.

Pulmonary congestion in HF activates stretch receptors and C-fibers, leading to irregular breathing patterns such as sighs, rapid shallow breathing, or periodic respiration. In addition, reduced cardiac output prolongs circulation time and heightens CO_2_ sensitivity, which can further destabilize respiration and result in Cheyne–Stokes breathing [39]. These disturbances collectively shorten RST and reflect worsening cardiopulmonary function [35].

## 11. How to Measure Respiratory Pattern

**Figure 4:**
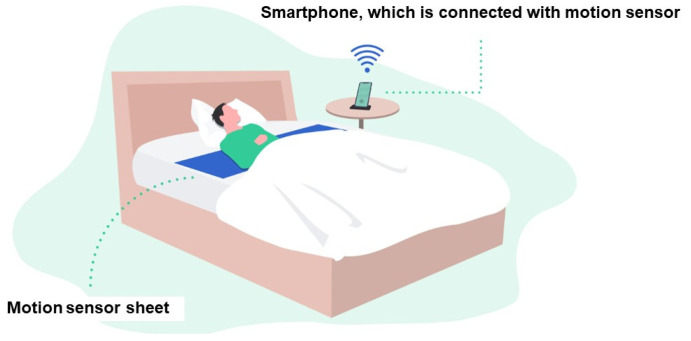
Contactless home monitoring system for RST measurement. RST is measured passively using a sensor placed beneath the mattress, which detects thoraco-abdominal motion without requiring wearable devices. Data are transmitted via a mobile phone to a cloud server and analyzed to calculate daily RST, enabling automated remote monitoring in heart failure patients.

RST is measured using a non-invasive, contactless sensor typically placed under the patient’s mattress (Figure 4) [40]. This sensor detects micro-movements associated with thoraco-abdominal breathing, capturing continuous respiratory signals without requiring any wearable devices or active patient participation. Such a feature is particularly advantageous for elderly patients.

Advanced signal processing algorithms analyze the data to identify the interval variabilities of the respiratory rhythm for one epoch (5 min). The interval variabilities of all epochs are averaged to calculate the RST for each night. Because the monitoring occurs passively and automatically in the home setting, RST offers high adherence and allows for daily longitudinal tracking, even in elderly or frail patients who may struggle with conventional monitoring methods. Importantly, RST measurements obtained from under-mattress sensors have been validated against gold-standard assessments, including polysomnography and established congestion markers, confirming their accuracy and clinical reliability.

## 12. RST as a Marker of Prognosis and Congestion

Recent studies have established that respiratory instability—quantified through RST—is a powerful and independent predictor of prognosis in patients with chronic HF. In an observational cohort study involving ambulatory patients with clinically stable HF, lower daytime RST values at rest were significantly associated with higher all-cause and cardiovascular mortality [35]. Patients with RST values below 20 were identified as being at particularly high risk, with markedly worse 5- and 10-year survival rates.

Multivariate analysis confirmed that RST provided prognostic information independent of sympathetic nerve activity and traditional prognostic markers such as plasma B-type natriuretic peptide and left ventricular ejection fraction.

**Figure 5:**
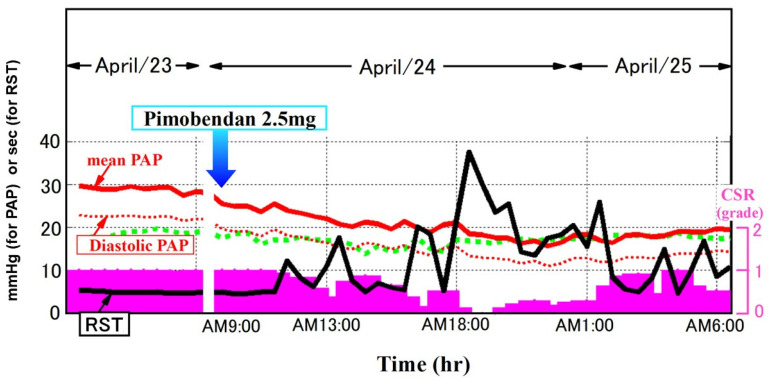
Temporal changes in RST during hospitalization for acute heart failure. In a representative patient, RST values gradually improved during treatment, including the initiation of oral inotropes, paralleling a decrease in pulmonary artery pressure and an amelioration of Chain–Stokes respiration (CSR). Reused with permission from Hidetsugu Asanoi.

Complementary evidence from a multicenter, prospective observational study further supports the pathophysiological relevance of RST in real-world clinical practice. In patients hospitalized for acute decompensated HF, RST levels significantly improved during clinical recovery, in parallel with the resolution of congestive signs, such as peripheral edema, weight gain, lung congestion, and elevated plasma B-type natriuretic peptide level (see a representative case in Figure 5).

Linear regression analysis revealed that changes in RST during hospitalization were closely correlated with markers of systemic and pulmonary congestion, accounting for over half of the observed variance in respiratory stability. Notably, patients whose clinical congestion failed to improve did not exhibit a meaningful increase in RST, underscoring its sensitivity to volume status. Another study also demonstrated that RST reflects lung congestion independently of blood oxygen concentration [41].

## 13. Remote Monitoring of RST and Its Potential Utility in Predicting Worsening HF

**Figure 6:**
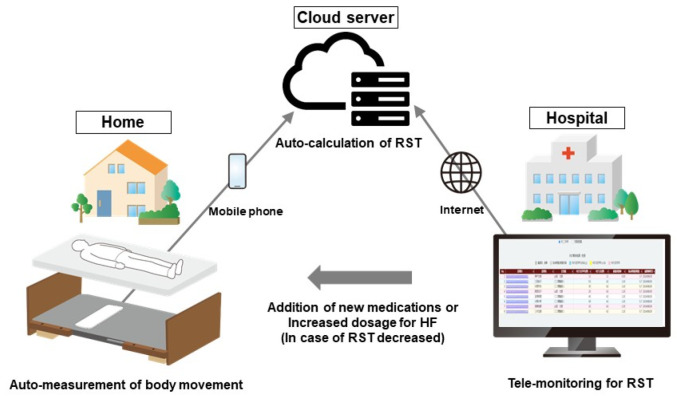
Telemonitoring architecture for real-time RST-based clinical decision support [42]. Home-based remote RST monitoring enables wireless data transmission to a centralized platform accessible to clinicians. Alerts for RST decline allow for timely therapeutic adjustment, such as diuretic up-titration, even in the absence of overt symptoms.

RST can be monitored longitudinally and non-invasively using a contactless sensor-based system installed beneath the patient’s mattress (Figure 4) [40]. This technology continuously captures micro-movements associated with thoraco-abdominal respiration during sleep, allowing automated, passive measurement of RST without the need for wearable devices or patient interaction. In the latest system, the collected data are wirelessly transmitted to a centralized server, where RST is calculated and displayed on a cloud-based clinical platform (Figure 6) [42]. This configuration enables daily remote monitoring of respiratory stability with minimal patient burden and high adherence, making it particularly suitable for the monitoring of elderly HF patients.

**Figure 7:**
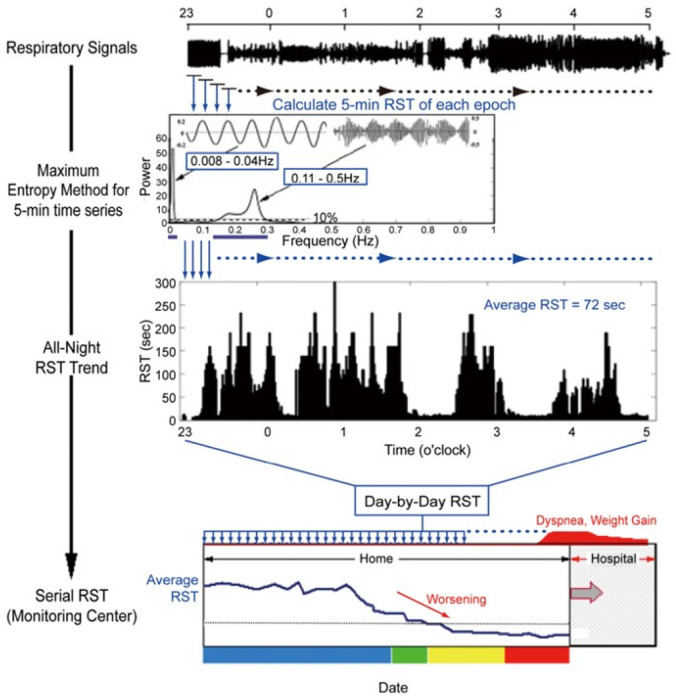
Predictive value of RST decline for impending heart failure readmission. Daily home RST trends in a representative patient show a sustained decline beginning several days before hospitalization. Notably, the drop in RST preceded the onset of clinical symptoms, such as dyspnea or weight gain, suggesting its utility for early detection of decompensation. Reused with permission [40].

The clinical utility of RST-based remote monitoring was recently demonstrated in a prospective, observational study involving patients with a history of hospitalization for HF [40]. In that study, daily RST trends were successfully tracked in the home setting following hospital discharge. Importantly, a sustained decrease in RST values was observed several days to weeks prior to clinically overt HF and related readmissions (Figure 7) [40]. In many cases, RST reduction preceded the development of typical symptoms such as dyspnea or weight gain, suggesting that RST captures subclinical physiological changes that herald decompensation.

Similar to prior studies, a threshold around 20 s was observed. This low inter-patient variability in this threshold enhances the specificity of RST declines as indicators of physiological deterioration. Notably, in patients whose RST declined by more than a predefined threshold, the majority required hospitalization within the following two weeks. Furthermore, when the declined RST level could be increased following aggressive therapeutic interventions, such as the up-titration of the diuretic dose, re-hospitalization due to HF seems to be avoided. However, it is uncertain whether the aggressive therapeutic intervention in patients with declining RST below its threshold has clinical benefits, given the lack of prospective interventional studies.

## 14. Ongoing Prospective Study Using RST-Guided HF Management

Building on the growing body of evidence that RST is a sensitive and potential early marker of HF decompensation [40], a novel interventional trial has been initiated to evaluate whether RST-guided management can improve clinical outcomes. Prior observational studies have demonstrated that declines in RST often precede symptomatic worsening and hospital readmission. Patients whose RST increased again beyond the threshold can avoid worsening HF. These findings have prompted the hypothesis that timely therapeutic intervention—triggered by a drop in RST, regardless of symptom presentation—may prevent full-blown decompensation and reduce the risk of hospitalization. We emphasize the importance of aggressive therapeutic intervention, even though the patients are asymptomatic, in the case of RST decline. Otherwise, the clinical decision on whether to intensify the therapy largely depends on other clinical factors, reducing the clinical implication of RST-guided early identification of worsening HF.

To test this hypothesis, a multicenter, historical-controlled trial entitled ITMETHOD-HF III (Intervention Trial Using RST-Guided Early Therapy for the Prevention of Heart Failure Readmission) is currently underway [42]. In the study, 80 ambulatory patients with a history of recent HF hospitalization are equipped with a non-contact RST monitoring system installed in their homes. As an intervention arm, medical therapy is adjusted based on predefined thresholds of RST decline (i.e., RST < 20 s). A historical control arm is used, in which standard care continues regardless of RST trends. The data of the control arm are derived from a baseline-matched previous study: ITMETHOD-HF II trial [42]. The analyses will compare the HF readmission rate between the intervention arm and the historical control arm during a 1.5-year observation period.

Importantly, the study protocol allows for therapeutic escalation—such as increasing diuretic dosage or optimizing GMDT—even in the absence of overt symptoms, when a decline in RST is detected. This strategy reflects a paradigm shift from reactive, symptom-driven care to preemptive, physiology-guided intervention.

The ITMETHOD-HF III trial represents the first rigorous attempt to evaluate whether RST-guided management can translate early physiological signals into actionable treatment strategies that improve long-term outcomes [42]. If successful, this approach may provide a practical and scalable model for home-based HF surveillance and intervention.

## 15. How to Improve RST Levels

Identifying a method to improve RST levels remains a future concern. RST probably declines due to the progression of pulmonary congestion and impaired systemic circulation [35]. In many HF out-patients, cardiac output should be relatively preserved, and a major contributor to worsening HF would be pulmonary congestion [43]. In most clinical scenarios with declined RST, up-titration of the diuretic dose may be recommended to improve RST levels [35].

**Figure 8:**
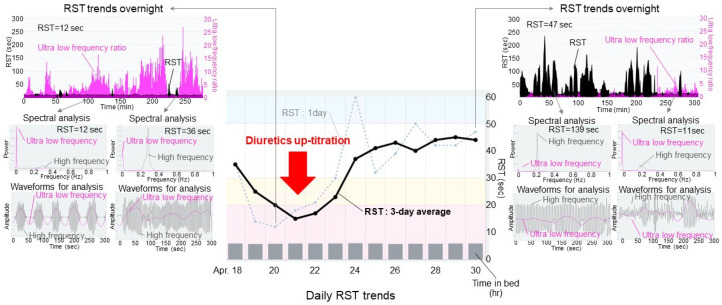
Clinical improvement in RST after diuretic optimization. In a patient with declining RST and predominant periodic breathing resembling Cheyne–Stokes respiration, up-titration of diuretics improved both RST and respiratory regularity.

Figure 8 shows a representative case, in which a declined RST improved by the up-titration of diuretics. When RST declined below 20 s, the respiratory pattern was irregular, accompanied by Cheyne–Stokes-like respiration. Following the up-titration of the diuretic dose, the RST level improved gradually, followed by an improvement in respiratory pattern regularity and the disappearance of Cheyne–Stokes respiration. In other cases, we have experienced RST improvement by the initiation of daytime adaptive servo-ventilation therapy, percutaneous mitral valve repair, and catheter ablation for atrial fibrillation, all of which should have ameliorated pulmonary congestion and improved cardiac output, stabilizing the respiratory pattern [44].

## 16. Future Concerns

Existing RST studies are encouraging but preliminary. Their strengths include non-invasive measurement, consistent thresholds, and correlation with congestion. However, most were small, single-country, observational or pilot studies with short follow-up, limiting generalizability [35]. Several challenges must be addressed before it can be widely adopted in routine clinical practice.

The clinical thresholds for intervention based on RST remain to be standardized. Although observational studies and pilot trials have suggested specific decline thresholds associated with adverse outcomes (i.e., RST < 20 s) [40], individualized cutoffs may be required depending on patient phenotype, comorbidities, and baseline respiratory stability. Further large-scale studies are needed to validate optimal intervention criteria and to ensure both sensitivity and specificity in diverse clinical settings.

The interpretation of RST in complex clinical scenarios remains an area of uncertainty. While RST primarily reflects pulmonary congestion and autonomic imbalance, it may also be influenced by comorbid respiratory conditions, such as sleep-disordered breathing, chronic obstructive pulmonary disease, or neuromuscular disorders [42]. Differentiating true HF-related deterioration from other causes of respiratory instability will be essential to avoid unnecessary interventions.

Integration into healthcare systems in real-world clinical practice remains challenging, requiring secure data transmission, real-time analysis, and clinical workflows that allow timely review and intervention. Effective implementation also depends on coordination among HF specialists, primary care providers, visiting nurses, and caregivers, particularly in aging societies where home-based care is increasingly important.

Reimbursement and cost-effectiveness remain key considerations. Although RST monitoring is non-invasive and passive, its economic impact must be justified through reductions in hospitalization, emergency visits, or healthcare utilization. Prospective cost-effectiveness analyses and health–economic modeling will play an important role in policy-making and regulatory approval.

Finally, patient and provider education will be crucial to promote adherence and appropriate use. Patients must understand the purpose of passive respiratory monitoring. At the same time, clinicians need guidance on how to interpret RST data and adjust therapy proactively.

Despite these challenges, the potential of RST-guided management to shift HF care from reactive to preventive is compelling. Ongoing clinical trials will clarify its role in patient selection, treatment algorithms, and health system integration. With appropriate validation and infrastructure, RST may become a cornerstone of next-generation remote HF management.

## 17. Conclusions

RST is a novel, non-invasive biomarker that enables the early detection of HF decompensation through remote monitoring. It reflects underlying congestion and autonomic imbalance, often preceding clinical symptoms. Current evidence supports its prognostic value, and an ongoing interventional trial is evaluating whether RST-guided therapy can reduce readmissions due to worsening HF. With further validation and system integration, RST may help shift HF care toward more proactive and preventive strategies.
